# Salivary Cortisol versus Serum Cortisol Levels in Asthmatic Children Treated with Inhaled Corticosteroid Alone or in Combination with Montelukast

**DOI:** 10.2147/JAA.S600420

**Published:** 2026-06-12

**Authors:** Atqah AbdulWahab, Rasha Abdeldaim Ali Alsiddig, Amal Al-Naimi, Mona Maarafiya, Ahmed Abushahin, Yahya Hani, Rajvir Singh, Jayakumar Jerobin, Ilham Bettahi, Mutasim Abu-Hasan

**Affiliations:** 1 Department of Pediatric Medicine, Division of Pulmonology, Sidra Medicine, Doha, Qatar; 2 Department of Laboratory Medicine and Pathology (DLMP), Hamad Medical Corporation, Doha, Qatar; 3 Department of Cardiology, Hamad Medical Corporation, Doha, Qatar; 4 Qatar Metabolic institute, Hamad Medical Corporation, Doha, Qatar

**Keywords:** asthma, children, inhaled corticosteroids, Montelukast, salivary cortisol, serum cortisol

## Abstract

**Objective:**

Inhaled corticosteroids (ICS) is the mainstay therapy of childhood asthma, but side effects related to hypothalamic-pituitary-adrenal (HPA) axis suppression is a concern, especially when combined with Montelukast. HPA suppression is assessed by morning measuring serum cortisol level, which is invasive and inconvenient in children. Salivary cortisol is a non-invasive alternative. We aim to correlate serum with salivary cortisol levels in asthmatic children, and assess the effect of treatment with ICS alone or ICS with Montelukast.

**Methods:**

Morning serum and salivary cortisol levels were measured in asthmatic children (7–12 years) who were treated with either ICS (IC group) or ICS plus Montelukast (ICM group). Salivary cortisol was also measured in age-matched healthy children (control group). We used Pearson coefficient to correlate between serum and salivary cortisol levels, and Student’s *t*-test or ANOVA for group comparisons as appropriate. We used multivariate regression analysis to evaluate predictors of salivary and serum cortisol levels.

**Results:**

A total of 150 children were recruited, 50 children in each study group. There was no difference between IC, ICM and control groups in age or BMI. Asthma groups had higher percentage of males (P= 0.01). We found positive correlation between serum and salivary cortisol levels (r = 0.29, p = 0.01). There was no difference in salivary cortisol levels between the 3 groups (7.30 ± 5.99 vs. 10.04 ± 11.6 vs. 10.7 ± 9.8, p = 0.76). There was no difference in serum cortisol levels between IC and ICM groups (249.03 ± 117.8 vs. 277.5 ± 123.9, p = 0.24). Age, gender, BMI, ICS dose and asthma severity did not affect serum or salivary cortisol levels.

**Conclusion:**

Salivary cortisol level correlates with serum cortisol in asthmatic children, but not different from level in controls, and not affected by treatment with ICS alone or with Montelukast.

## Introduction

Asthma is the most common chronic airway inflammatory disease in children and adolescents, with progressively increasing prevalence rates.^1^ The goals of managing asthma, according to International guidelines are controlling symptoms, improving lung function, and minimizing medication side effects.^2^ Inhaled corticosteroids (ICS) are the cornerstone class of medications for asthma treatment in both children and adults.^3^

ICS achieve therapeutic effect through binding to glucocorticoid receptors in the airways and suppressing inflammation. The use of inhaled ICS is preferred over systemic steroids to control asthma because of their minimal side effects considering that the systemic availability of ICS is far less than that of oral or IV corticosteroids.^3^ However, ICS can still access blood stream and have deleterious side effects, especially related to glucose metabolism, decrease in height velocity growth, blood pressure control and suppression of the hypothalamic-pituitary-adrenal (HPA) axis. ICS can suppress the HPA by reducing corticotrophin (ACTH) production by anterior pituitary gland, which in turn reduces serum cortisol secretion by the adrenal gland.^4–7^ Stress related to asthma symptoms and asthma exacerbations can potentially suppress serum cortisol production regardless of treatment with steroids.^8^,^9^ Treatment of asthma with inhaled steroids, on the other hand, are associated with significant cortisol suppression as confirmed by several studies performed in both children and adults.^4^,^10^

Therefore, monitoring early morning serum steroids levels can help prevent ICS related side effects, including HPA suppression.^11^ However, obtaining early morning serum steroids levels in children is invasive and inconvenient. Also, serum cortisol level does not accurately reflect adrenal suppression. The standard tests of serum cortisol levels measure total serum cortisol, which includes both the protein bound cortisol as well as the free cortisol, the latter being the biologically active form. For these reasons, measuring salivary cortisol levels has been advocated as an alternative to serum cortisol measurement since it is painless, and relatively stress-free non-invasive method of specimen collection, especially in children, can be obtained at home or at school, and measure only free and biologically active form of cortisol. Moreover, salivary cortisol tends to correlate strongly with serum cortisol.^12^,^13^

Several studies advocate use of salivary cortisol in assessing adrenal suppression using ACTH stimulation tests in children.^14^,^15^ However, the diagnostic value of measuring salivary cortisol in assessing HPA suppression in children with asthma specifically is not well established.^16^

Studies correlating salivary cortisol with serum cortisol levels in children with asthma are lacking. Also, the effect of asthma severity or ICS treatment on the degree of HPA suppression using salivary cortisol levels are unknown. Because of the known HPA suppressive effect of asthma itself as well as inhaled steroids on serum cortisol levels, salivary cortisol levels are expected to be also low in asthmatic children, especially in patients with severe asthma who are treated with high dose ICS.

Furthermore, the potential interaction between ICS and non-steroidal anti-inflammatory medications such as leukotriene receptor antagonists (LTRA) has not been previously explored. It is unclear whether adding LTRA, such as Montelukast, to ICS improves or worsens HPA suppression. LTRA medications have the additive effect of reducing airway inflammation and preventing bronchoconstriction.^17^ The international guidelines recommend their use as monotherapy for patients with mild persistent asthma or as combination therapy with ICS to avoid increasing ICS dose.^17^ In this case, LTRA can potentially ameliorate the effect of ICS on HPA suppression and improve salivary cortisol levels. On the other hand, Montelukast is known to inhibit the cytochrome P450 (CYP2C8) enzyme activity in the liver, which can potentially decrease ICS metabolism.^18^ In this case, Montelukast may result in worsening HPA suppression and lower salivary cortisol levels in patients treated with ICS and Montelukast combination.

In this study, we aim to correlate early morning salivary cortisol levels with early morning serum cortisol levels in children with asthma. We also aim to compare salivary cortisol between children with asthma and healthy children, and asses the effect of asthma severity and ICS dose used on serum/salivary cortisol levels. Finally, we aim to assess the effect of treating asthmatic children with ICS versus ICS/Montelukast combination on serum and salivary cortisol levels.

## Materials and Methods

### Study Design and Population

This is a cross sectional study of 3 groups: 1- children with asthma who were treated with ICS for at least 3-month period **(IC group**), 2- children with asthma who were treated with inhaled steroids plus Montelukast for at least 3-month period (**ICM group**), and 3- age matched healthy children (**control group**). Children with asthma were recruited during visits to the pediatric pulmonary outpatient clinic at Sidra Medicine (Doha, Qatar) between January 2022 and May 2024. Siblings of asthmatic children were also recruited in the control group after parents’ consent and child’s assent. The study was conducted in compliance with the Helsinki declaration. Informed written consent was obtained from the parents, and informed written assent was obtained from the children. The study was approved and monitored by the Institutional Review Board (IRB No. 11896792).

Inclusion criteria for IC and ICM groups were as follows: (1) age between 7 and 12 years old, (2) prepubertal status according to Tanner staging, (3) confirmed diagnosis of asthma by the treating physician and in accordance with the American Thoracic Society (ATS), Global Initiative for Asthma (GINA), and International Study of Asthma and Allergies in Childhood (ISAAC) guidelines for asthma diagnosis, and (4) treatment with daily ICS (IC group) or daily ICS plus Montelukast (ICM group) for at least three months period prior to enrollment. Inclusion criteria for the control group were as follows: (1) age between 7 and 12 years, and (2) healthy with no known acute or chronic diseases.

Study exclusion criteria were as follows: (1) asthma exacerbation within the past three months requiring increase in baseline ICS dose, oral steroids or IV steroids, (2) chronic disease or comorbidity that could affect adrenal function (eg., interstitial lung disease, cystic fibrosis, primary ciliary dyskinesis, bronchiectasis, congenital heart disease, diabetes, and immune deficiency)., (3) use of systemic corticosteroids in the past three months, (4) use of topical corticosteroids or intranasal corticosteroids in the past three months, and (5) use of other medications that can interfere with cortisol metabolism (ie. azithromycin).

At recruitment, the following subject data were obtained: age, gender, growth parameters (weight, height, BMI), clinical symptoms in the past three months, pulmonary function test results, medications used, compliance with medications (as reported by the patient or parent). Asthma was considered mild if FEV1 was ≥ 80%, moderate if FEV1 was > 60 but < 80%, and severe disease if FEV1 was < 60%.

ICS dose was documented as actual fluticasone dose if patient was taking fluticasone or as fluticasone equivalent if patient was taking other ICS formulations. Fluticasone equivalence of ICS medications was based on their fluticasone-relative potency. For example, if patient was taking budesonide, the ICS dose was calculated based on the ratio of clinical potency of budesonide relative to fluticasone propionate which is 1:2. The ICS dose used by patients was considered low if fluticasone dose or its equivalent were <200 mcg/day, medium if fluticasone dose or its equivalent were 200–400 mcg/day, and high if fluticasone dose or its equivalent were >400 mcg/day.^19^

### Anthropometric Measurements and Lung Function Measurements

Anthropometric measurements, and lung function test results were performed during first study visit. Weight was measured using digital electronic scale, and height was recorded using a stadiometer. Body mass index (BMI) was calculated as weight (kg) / height (m^2^). The standard deviation (Z) scores for weight, height, and BMI were calculated using WHO data for age and gender.

Lung function testing was conducted according to ATS/ERS guidelines, using a Jaeger MS-PFT device (CareFusion, Leibniz Strasse, Germany). Predicted values were calculated based on reference data from the Global Lung Initiative (GLI).^20^ Only tests that met ATS acceptance criteria were included. The best of three acceptable measurements for forced vital capacity (FVC), forced expiratory volume in the first second (FEV1), FEV1/FVC ratio and FEF 25-75% predicted were recorded.

### Serum and Saliva Collection and Biochemical Analysis

Morning saliva and serum cortisol level during the second study visit which was planned during the weekend following first study visit. Saliva and serum samples were collected simultaneously from the asthmatic children (IC and ICM groups), but saliva samples only were collected from the children in the control group. All samples were obtained between 8:00 and 9:00 a.m. after an overnight fast. Saliva was collected using a sterile Salivette cotton swab. Subjects were asked to chew the swab for one minute to ensure it was adequately saturated with saliva, without touching the swab with hands. Samples were then transported to the research laboratory at Hamad Medical Corporation (HMC), Doha, Qatar, within one hour of collection, for analysis. Samples were immediately centrifuged at 2000 x g for 10 minutes, and then stored at −80°C. Salivary cortisol levels were measured using a competitive enzyme immunoassay (Cortisol Parameter Assay Kit, Catalog #: KGE008B). Serum cortisol analysis was performed using a competitive electrochemiluminescence immunoassay (ELICA; Assay Kit: Elecsys Cortisol II, Instrument / Platform: Cobas e 801 (Roche Diagnostics).

### Sample Size

Sample size calculation was based on salivary cortisol levels obtained in previous study comparing salivary cortisol between children with asthma and healthy controls,^8^ Based on that study, the sample size required to detect a mean difference in salivary cortisol level of 0.1 nmol/L between the IC, ICM, and control groups, with 95% confidence interval between −2.2 and 1.9 nmol/L, 80% power, and 5% type I error rate, was 132 subjects (44 per group). To account for potential dropouts, the sample size was increased by 10%, resulting in a total of 150 participants (50 per group).

### Statistical Analysis

Frequencies and percentages were used to describe categorical variables, while means and standard deviations or medians and interquartile range were used to describe continuous variables based on their normality of distribution. The Shapiro–Wilk and Kolmogorov–Smirnov tests were used to assess data normality. Based on the distribution, appropriate parametric or non-parametric statistical tests were applied.` For normally distributed data, Student’s *t*-test and one-way ANOVA were used to compare between groups as appropriate. For non-normally distributed data, Mann–Whitney *U*-test or Kruskal–Wallis test were used for group comparisons as appropriate. Regression analysis was conducted to evaluate the potential predictors of salivary cortisol level in the entire cohort (age, gender, BMI and asthma diagnosis), and the potential predictors of serum levels in the asthma groups (ICS dose, asthma severity, FEV1% predicted, FVC% predicted, FEV1/FVC % predicted and FEF 25–75% predicted). P-value < 0.05 (two-tailed) was considered statistically significant. All analyses were performed using SPSS Statistics for Windows, Version 29.0 (Armonk, NY: IBM Corp).

## Results

A total of 150 children were included in the study: 50 in IC group (37 males, 13 females), 50 in ICM group (38 males, 12 females), and 50 in control group (25 males, 25 females). There was no significant difference between the three groups in age, weight, height, or BMI Z-scores. However, the control group had a significantly lower male-to-female ratio compared to ICS and ICM groups (P = 0.01) (Table 1).

**Table 1:** Comparisons Between Group of Asthmatic Children Treated with ICS (IC Group), Asthmatic Children Treated with ICS and Montelukast (ICM Group), and Healthy Subjects (Control Group)

Variables	Control Group (n=50)	Inhaled Steroids Group (ICS) (n=50)	Inhaled Steroids Plus Montelukast Group (ICM) (n=50)	P value
**Age (years) (mean ± SD)**	9.20 ±1.85	9.24 ± 1.61	9.60 ± 1.65	0.44
**Male Gender, n (%)**	25 (50%)	37 (74%)	38 (76%)	0.01
**Salivarycortisol nmol/L (mean ± SD)**	7.30 ± 5.99	10.70 ± 9.79	10.04 ± 11.55	0.17

The ICM group had higher proportion of children with positive family history of allergies compared to the IC group (Table 2). Additionally, the ICM group had significantly lower pulmonary function compared to the IC group, including FEV1% predicted, FEV1/FVC ratio, and FEF25–75% predicted (Table 2).

**Table 2:** Comparisons Between Asthmatic Children Treated with ICS (IC Group) and Asthmatic Children Treated with ICS and Montelukast (ICM Group)

Variables	Inhaled Steroid Group (IC) (n=50)	Inhaled Steroids Plus Montelukast Group (ICM) (n=50)	P-value
Age (year) (mean ± SD)	9.2±1.6	9.6±1.7	0.82
Boys n (%)	37(74%)	38 (76%)	0.82
Allergic Rhinitis (n, %)	10 (20%)	16 (32%)	0.21
Family history of atopy (n, %)	24 (38%)	39 (62%)	0.00
Eczema (n, %)	5 (10%)	11 (22%)	0.11
Weight (mean ± SD)	38.1 ± 11.1	39 ± 11.3	0.67
Weight *Z* score (mean ± SD)	0.6 ± 1.3	0.7 ± 1.18	0.64
Height (mean ± SD)	138.2 ± 11.4	140 ± 11.4	0.4
Height *Z* score	0.13 ± 1	0.4 ± 1	0.26
BMI (mean ± SD)	19.5 ± 4.2	19.6 ± 3.8	0.88
BMI *Z* score	0.6 ± 1.3	0.62 ± 1.2	0.85
Total IgE (mean ± SD)	691.3 ± 728.7	729.9 ± 745.5	0.79
FVC% Predicted (mean ± SD)	97.9 ± 11.30	94.7 ± 11.6	0.19
FEV1% Predicted (mean ± SD)	94.4 ± 13.3	86.4 ±11.8	0.003
FEV1/FVC (mean ± SD)	84.2 ± 5.8	79.8 ± 5.8	0.003
FEF 25–75% Predicted (mean ± SD)	79.5 ± 21.7	66 ± 16.3	0.001
Serum cortisol in nmol/L (mean ± SD)	277.5 ± 123.9	249 ± 117.8	0.24
Salivary cortisol in nmol/L (mean ± SD)	10.7 ± 9.8	10.04 ±11.6	0.76

**Abbreviations**: BMI, Body Mass Index; FVC, Forced Vital Capacity; FEV1, Forced Expiratory Volume in 1 second; FEF 25–75%, Forced Expiratory Flow between 25% and 75% of FVC.

Majority (80%) of asthmatic children were treated with fluticasone MDI using a spacer, or with fluticasone plus Montelukast. The median inhaled fluticasone dose, or equivalent, was 250 mcg/day (range: 100–500 mcg/day). Of the 100 asthmatic children, 27 received low dose, 65 medium dose, and 8 high dose ICS. Regression analysis showed age was the only significant predictor of high salivary cortisol level in the entire study population. Gender, weight, height and asthma diagnosis were not significant predictors.

**Figure 1:**
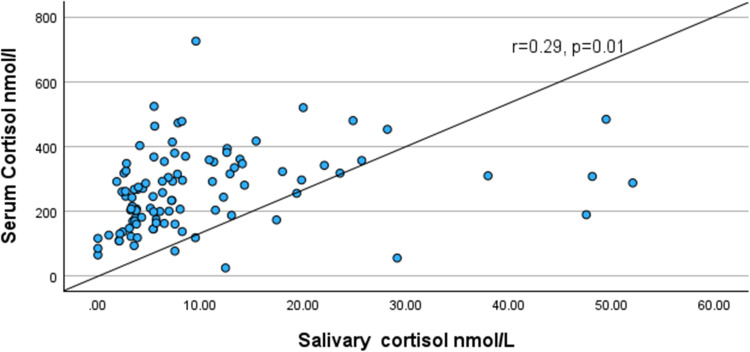
Correlation between serum and salivary cortisol in asthmatic children.

A positive correlation between serum and salivary cortisol levels was observed in the asthmatic groups (r = 0.29, p = 0.01) (Figure 1). However, there was no significant difference in salivary cortisol levels between IC, ICM and control groups (p = 0.17) (Table 1). Also, there was no difference in serum cortisol levels between IC and ICM asthma groups (p = 0.24) (Table 2).

**Figure 2:**
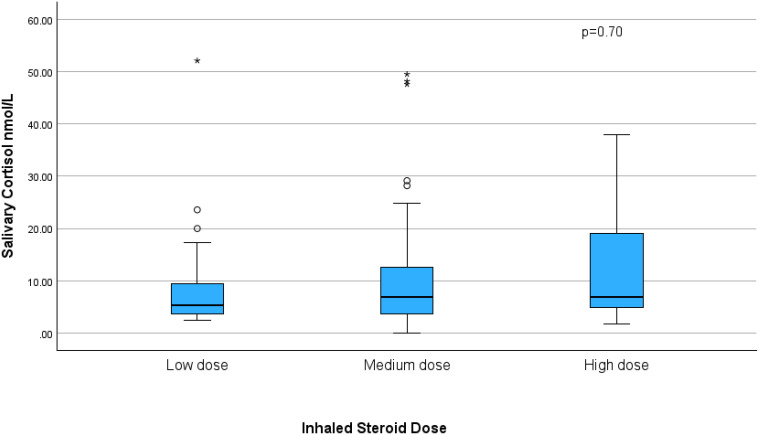
Salivary cortisol level in all asthmatic children according to inhaled steroid dose received (low dose: <200 mcg/daily, medium dose: 200–400 mcg/daily, High: >400 mcg/daily).

**Note**: ° = outliers and * = extreme outliers.

**Figure 3:**
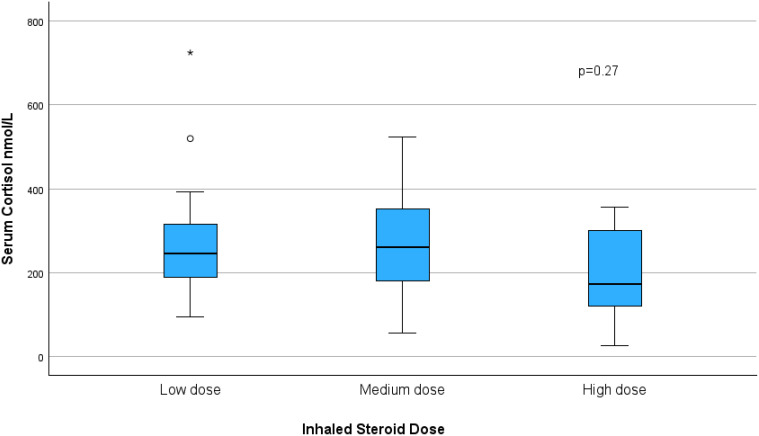
Serum cortisol level in asthmatic children according to inhaled steroids doses (low dose: <200 mcg/daily, medium dose: 200–400 mcg/daily, High: >400 mcg/daily).

**Note**: ° = outliers and * = extreme outliers.

**Figure 4:**
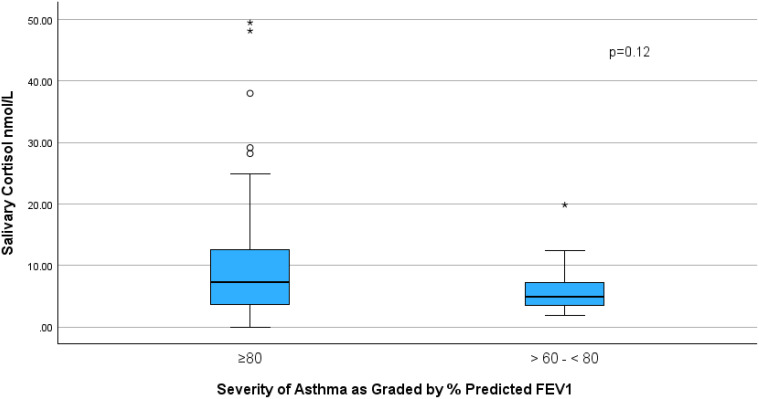
Salivary cortisol level in asthmatic children according to their lung function testing results (FEV1% predicted ≥ 80% predicted vs >60%but<80% predicted).

**Note**: ° = outliers and * = extreme outliers.

Majority of the children with asthma (89%) were able to perform spirometry. Seventy-one patients had mild disease, 18 patients had moderate disease and none (0%) had severe disease. There was no significant difference in serum or salivary cortisol levels between children who were treated with low dose, medium dose, or high dose ICS (Figures 2 and 3). In addition, there was no significant difference in salivary cortisol levels between mild and moderate asthma groups (Figure 4). Regression analysis showed that ICS dose, asthma severity, FEV1 % predicted, FVC% predicted, FEV1/FVC% predicted and FEF 25–75% predicted did not significantly predict salivary or serum cortisol levels in the asthmatic groups.

## Discussion

Our objectives in this study were to evaluate the diagnostic value of salivary cortisol level as a marker of HPAaxis suppression in children with asthma who are treated with inhaled steroids or combination of ICS and Montelukast, and compare it to conventional measurement of serum cortisol level. We found weak but significant correlation between salivary cortisol and serum cortisol levels. Previous studies demonstrated stronger correlation between salivary and serum cortisol levels, and have advocated for the use of salivary cortisol as a non-invasive measure of HPA axis suppression using ACTH stimulation test.^21^,^22^ However, the use of salivary cortisol level as a marker of adrenal suppression in asthmatic patients using ICS, particularly in children is not well established.^16^

Our data suggest that the use of salivary cortisol in children with asthma should be interpreted with caution because of the week correlation found. This week correlation could be attributed to variation related to sampling technique and possible interference of inhaled steroids in salivary cortisol testing method, especially when Immunoassays, unlike Liquid Chromatography-Tandem Mass Spectrometry (LC-MS/MS), are prone to cross-reactivity with synthetic glucocorticoids, leading to false-positive or false-negative results.^23^

Contrary to previous studies, our study showed no significant HPA-axis suppression, as measured by salivary and serum cortisol levels, in children with asthma compared to healthy controls. Some studies showed that children with asthma have experienced HPA-axis suppression after taking high dose as well as low dose ICS.^24^,^25^ Other studies showed no effect of inhaled steroids on adrenal suppression.^26^ In a study by Priftis et al, 20% of asthmatic children treated with low to moderate doses of inhaled budesonide mild adrenal suppression was demonstrated using low-dose synacthen test (LDST).^27^ On the other hand, previous studies showed reduced baseline adrenal function in children with asthma even before starting ICS treatment suggesting that HPA-axis suppression is related to asthma itself rather than ICS therapy.^9^

Based on our study findings, routine monitoring of serum or salivary cortisol may not be warranted for children receiving ICS treatment. However, these findings may not be generalizable to children with asthma who are treated with very high doses of ICS over extended periods of time, or to children with obvious clinical symptoms of HPA-axis suppression.

Our study also showed that adrenal suppression, as measured by serum and salivary cortisol levels, was not affected by the dose of ICS used. We found no differences in salivary or serum cortisol levels among children treated with low, medium, or high doses of ICS. The small number of patients in each subgroup and variations in adherence to medications among patients could contribute to this negative result. Previous studies showed low serum cortisol levels even in children treated with low dose ICS, however, a systematic review study suggests that ICS doses generally have little to no impact on plasma or urine cortisol levels.^28^

No previous studies have examined the potential effect of adding oral Montelukast to ICS treatment on salivary or serum cortisol levels in children with asthma. In this study, we found no differences in salivary or serum cortisol levels between children who were treated with ICS alone and those who were treated with ICS plus Montelukast. Despite the higher ICS dose used in patients treated with ICS and Montelukast combination, morning salivary and serum cortisol levels were similar to ICS only group, which suggest that adding Montelukast does not contribute to additional HPA-axis suppression.

The study has limitations that can affect the reliability of its overall conclusions. The dependence on patient and/or parent reports to assess adherence to ICS/Montelukast therapy and the duration of treatment, makes the data susceptible to recollection bias. Also, the small number of patients in each study group and subgroup can increase the likelihood of type II error. Finally, the short duration of ICS alone or ICS plus Montelukast treatment, and the small percentage of patients with severe asthma or those received very high ICS doses, limits the generalizability of our findings to these populations.

## Conclusions

Salivary cortisol level correlated with serum cortisol levels in children with asthma. However, the correlation is not strong enough to justify its use as an alternative to serum cortisol level in clinical settings. Also, there is no evidence of suppression of either salivary or serum cortisol levels in children with asthma who were treated with ICS alone or ICS plus Montelukast. These findings argue against the routine use of salivary or serum cortisol level in children with asthma. Further research is needed to explore the effects of ICS or ICS plus Montelukast on salivary cortisol levels in children receiving high doses of ICS for extended periods of time.
